# Exposure to Arboviruses in Cattle: Seroprevalence of Rift Valley Fever, Bluetongue, and Epizootic Hemorrhagic Disease Viruses and Risk Factors in Baringo County, Kenya

**DOI:** 10.3390/pathogens13080613

**Published:** 2024-07-24

**Authors:** Tatenda Chiuya, Eric M. Fèvre, Noah O. Okumu, Abdullahi M. Abdi, Sandra Junglen, Christian Borgemeister

**Affiliations:** 1 Centre for Development Research (ZEF), University of Bonn, Genscherallee 3, 53113 Bonn, Germany; cborgeme@uni-bonn.de; 2 International Livestock Research Institute, Old Naivasha Road, P.O. Box 30709, Nairobi 00100, Kenya; eric.fevre@liverpool.ac.uk (E.M.F.); n.okumu@cgiar.org (N.O.O.); abdullahimohaa4459@gmail.com (A.M.A.); 3 Institute of Infection, Veterinary and Ecological Sciences, University of Liverpool, Liverpool L69 3BX, UK; 4 Institute of Virology, Charité-Universitätsmedizin Berlin, Corporate Member of Free University Berlin, Humboldt-University Berlin and Berlin Institute of Health, 10117 Berlin, Germany; sandra.junglen@charite.de

**Keywords:** cattle, Rift Valley fever, bluetongue, epizootic hemorrhagic disease, inter-epidemic, seropositivity

## Abstract

Rift Valley fever virus (RVFV) causes disease outbreaks in livestock and humans; however, its inter-epidemic circulation is poorly understood, similar to other arboviruses affecting cattle such as bluetongue virus (BTV) and epizootic hemorrhagic disease virus (EHDV). Serum samples were collected in Baringo County, Kenya from 400 cattle, accompanied by a risk factor questionnaire. Serological tests were then conducted to determine the exposure of cattle to RVFV, BTV, and EHDV. RVFV, BTV, and EHDV IgG seroprevalence rates were 15.5%, 91.5%, and 91%, respectively. Seropositivity for RVFV, BTV, and EHDV was significantly higher in adult cattle, as well as in females for RVFV. Cattle with herd owners aged between 30–39 years were less likely to be seropositive for RVFV compared to those with owners over the age of 60 years. High seroprevalence of BTV and EHDV in cattle indicates significant exposure and the subclinical circulation of these viruses, presenting a risk of outbreaks to sheep and naïve cattle. Moreover, the detection of RVFV-seropositive young cattle born after the last reported outbreak suggests inter-epidemic circulation of the virus. Overall, monitoring these arboviruses in cattle is crucial in understanding their distribution and seroprevalence during inter-epidemic periods.

## 1. Introduction

Livestock production in sub-Saharan Africa (SSA) is hampered by several factors including feed and water availability and disease occurrence. These factors are likely to be exacerbated by climate change, particularly in arid and semi-arid ecosystems [1,2]. Livestock diseases pose much higher risks in SSA than in any other region because of the tropical climate, often inadequate veterinary services, and uncontrolled animal movement [3]. In SSA, diseases are responsible for mortality rates of about 7% and 21% in adult cattle and calves per year, respectively [4].

Rift Valley fever (RVF), caused by the Rift Valley fever virus (RVFV) (genus *Phlebovirus*: family *Phenuiviridae*), is a hemorrhagic disease affecting both livestock and humans [5]. It is transmitted to livestock and humans through infected mosquito bites. Other transmission routes include direct contact with shed abortus and infectious fluids, as well as the consumption of raw milk and blood from infected animals [6]. The clinical signs in cattle include fever, abortion, loss of appetite, and decreased milk production, while in humans, a non-specific febrile illness is common [6]. The disease is endemic in SSA and several outbreaks have been reported in Kenya. In the last major outbreak of 2006/2007, Baringo County in the northwest of the country was one of the heavily affected regions, with 88 human and 36 livestock cases confirmed [7]. While the RVF outbreak dynamics and associated losses have been clearly documented [8], very limited information is available on its inter-epidemic (IEP) transmission and effects on livestock health. Currently, vertical transmission of the virus in *Aedes* spp. mosquitoes, horizontal transmission in susceptible livestock/wildlife, intermediate rainfall, and recurrent introduction of the virus from ‘hotspot’ regions are some of the proposed factors responsible for the maintenance of RVF during IEPs [9].

Bluetongue (BT) and epizootic hemorrhagic disease (EHD) are caused by the bluetongue virus (BTV) and epizootic hemorrhagic disease virus (EHDV), respectively, both orbiviruses from the family *Sedoreoviridae* (formerly *Reoviridae*) [10]. Currently, 29 serotypes of BTV and 11 other atypical groupings circulate worldwide [11,12,13,14]. Unlike RVFV, BTV and EHDV are both transmitted by biting midges, primarily *Culicoides imicola* Kieffer (Dipt.: Ceratopogonidae) in Africa [15]. BT is endemic in Africa, where it affects domestic and wild ruminants; however, outbreaks are usually reported in exotic sheep breeds. The disease in sheep is characterized by fever, facial edema, lacrimation, and oral/nasal hemorrhage [16]. In susceptible flocks, morbidity can be as high as 100% while mortality rates range from 2–30% [17]. In cattle, BT is usually subclinical, hence it can circulate undetected; however, some serotypes such as BTV-8 have produced clinical disease in cattle during outbreaks in northwestern Europe [18]. While BT outbreaks usually have a more devastating effect [19], the omnipresent subclinical disease is also associated with loss of body weight, a decrease in milk production and reproductive performance, and livestock trade restrictions for infected herds [20]. A longitudinal study in western Kenya demonstrated that by 1 year of age, 94% of calves had been exposed to BTV and had circulating antibodies, indicating a high prevalence of the virus [21].

EHD was first described in New Jersey in 1955 in white-tailed deer, where it shows an overt clinical syndrome characterized by per-acute death, fever, oral ulceration, edema, and lameness [22]. The disease is usually subclinical in cattle, with clinical signs such as fever, inappetence, decrease in milk production, and nasal erosions. For example, in Israel, it was estimated that an EHDV seroprevalence of >80% is associated with a 2% loss in the annual production of dairy cattle [23]. Seven serotypes of EHDV circulate in several countries in Africa, Asia, the Middle East, and North and South America [24]. Outbreaks have been reported in Turkey [24], Israel [23], Spain [24], and Tunisia [25].

Baringo County lies in the postulated BTV enzootic area and is also an arbovirus hotspot [26]. This is highlighted by previous RVFV outbreaks and the circulation of other zoonotic arboviruses [7,27,28,29]. Additionally, occasional flooding events from the Baringo and Bogoria Lakes and the high annual temperatures create a conducive environment for the proliferation of mosquito and culicid vectors. In previous surveys in the region, only a small percentage of livestock owners were aware of RVFV and not of any other arboviruses [30,31]. It is possible that several arboviruses affect cattle in this region. Thus, the objective of our study was to improve the knowledge of IEP circulation of RVFV and the subclinical occurrence of BTV and EHDV in cattle in Baringo County, as well as to contribute data to a better understanding of the distribution of these viruses in diverse ecosystems in the Horn of Africa.

## 2. Materials and Methods

### 2.1. Study Site and Design Characteristics

**Figure 1:**
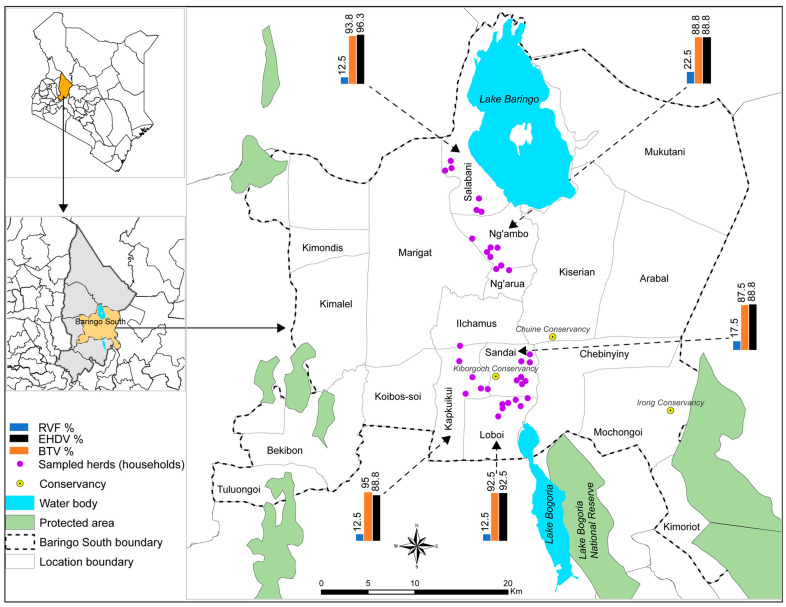
A map of Baringo County, Baringo South sub-County, and the locations and households where cattle were sampled. Bar charts show the seroprevalence of RVFV, BTV, and EHDV in the five locations. Water body, country, and county boundary data were downloaded from the World Resources Institute (https://www.wri.org/resources/data_sets (accessed on 27 April 2019) [37]. The map was developed using ArcGIS Software Version 10.2 (http://desktop.arcgis.com/en/arcmap (accessed on 27 April 2019) [38].

The study was carried out in Baringo County in the Kenyan Rift Valley (Figure 1). Five locations were selected on the basis of having previous reports of RVF cases during past outbreaks, arbovirus circulation in cattle and humans, and proximity to community conservancies and lakes. The five locations were Sandai, Kapkuikui, Loboi, Ng’ambo, and Salabani. Annual precipitation in the Baringo highlands has been recorded between 1000–1500 mm and 300–700 mm in the low-lying areas, with peak precipitation experienced in April and November. The climate is hot and semi-arid, with temperatures ranging from 10 °C in the highlands to 35 °C in the lowlands [32].

This cross-sectional study was carried out between 1–14 April 2023. Sample size calculations were based on Cochran’s formula for sample size determination [33] using a previously reported seroprevalence of RVFV (6.1%) in Baringo County [34]. Initially, an unadjusted sample size of 86 was calculated and then adjusted to 320 cattle after considering the intra-herd clustering coefficient (ICC = 0.3) [35] and a calculated design effect of 3.7 according to Alimohamadi et al., 2019 [36]. We then increased the final sample size to 400 to cover for non-responses, with 80 animals being sampled from each of the 5 locations. Initially, we planned to randomly select 8 herds in which 10 animals will be randomly sampled per location. However, information from a recent survey on arbovirus knowledge showed that few households had cattle [30]. These herds were therefore conveniently selected and between 5–23 cattle that were at least 6 months old were sampled per herd.

### 2.2. Sample and Data Collection

All the cattle in this study were sampled from smallholder livestock farmers at the owners’ households. Signalment and history of the animal were taken and a brief clinical examination was performed before blood collection. Using a brief questionnaire (File S1), the collected information included age, sex, breed, rectal temperature, previous vaccinations, body condition score, presence/absence of ticks, history of sickness, and clinical signs. Blood was collected into 10 mL plain tubes using an 18-gauge rubber-capped needle (BD, Franklin Lakes, NJ, USA). The plain tubes were spun in a centrifuge (Eppendorf, Hamburg, Germany) at 2000× *g* for 10 min to separate the serum, which was aliquoted into cryovials. Serum samples were then stored in liquid nitrogen. Demographic and socio-economic data were available for each household from a previous survey on RVF [30]. For the newly introduced 14 households, the previously used questionnaire (File S2) was administered during cattle sampling.

### 2.3. RVFV, BTV, and EHDV Competitive ELISA

Testing for antibodies against BTV and EHDV in serum was carried out using an ID Screen^®^ BT indirect competitive ELISA kit (IDvet, Grabels, France) and an ID Screen^®^ EHDV indirect competitive ELISA kit, respectively (IDvet, Grabels, France). For RVFV, an ID Screen^®^ RVFV indirect competitive ELISA kit (IDvet, Grabels, France) that detects both RVFV IgG and IgM antibodies was used. Testing and interpretation of the results were conducted according to the manufacturer’s instructions. The microplates for RVFV were coated with a virus nucleoprotein antigen, while for BTV and EHDV, they were coated with recombinant BTV VP7 and EHDV VP7 protein antigens, respectively. Optical density (OD) values were read at 450 nm in an ELISA microplate reader (Synergy, BioTek, Winooski, VT, USA). For each sample, a competition percentage (S/N%) was calculated as follows: S/N% = (OD_sample_/OD_negative control_) × 100. Samples presenting an S/N percentage (S/N%) (i) greater than or equal to 40% were negative, (ii) greater than 30% and less than 40% were doubtful, and (iii) less than or equal to 30% were positive. Doubtful samples were regarded as negative. The diagnostic sensitivity and specificity for the RVFV kit are previously reported to be 98% and 100%, respectively [39]. For the BTV kit, sensitivity and specificity are quoted at 100% and 99.3%, respectively [40], while for EHDV it is between 90−100% and 100%, respectively. An exclusivity of 98% with respect to BTV has been reported for the EHDV kit [41].

### 2.4. RVFV IgM Antibody Capture ELISA

Testing for IgM antibodies against RVFV was performed using the ID Screen^®^ RVFV IgM Capture kit (IDvet, Grabels, France) only for those samples that contained anti-RVFV IgG antibodies. This is because the initial RVFV competitive ELISA kit detects both IgG and IgM antibodies against RVFV. The testing procedure and interpretation of results was also performed according to the manufacturer’s specifications. After reading the optical density of each well at 450 nm, the percentage of the ratio of sample and positive control (S/P%) was calculated as follows: S/P% = (net OD_sample_/net OD_postive control_) × 100. Samples presenting an S/P percentage (S/P%) (i) less than or equal to 40% were negative, (ii) between 40% and 50% were doubtful, and (iii) greater than or equal to 50% were positive. The doubtful samples were considered to be negative.

### 2.5. Statistical Analysis

A logistic regression model was fitted to the data to determine the association between the independent and dependent variables. The independent variables included owner/household data (socio-economic index, age, gender, education level, occupation, history of RVF, distance to conservancy, tropical livestock units, and knowledge of RVF) and animal level data (age, body condition score, sex, and history of sickness). The dependent variable was seropositivity to RVFV, EHDV, and BTV. We also examined if there was any association between seropositivity to one virus and the other viruses under study. Univariate analysis was performed and all the factors with a *p*-value < 0.2 were included in the multivariate models. Multivariate analysis was carried out with *stepAIC* function (reverse) to derive the final models. All analyses were carried out in R^®^ version 4.3.1 [42]. Multicollinearity among all the variables was checked using the generalized variance inflation factors (GVIFs) for both continuous and categorical variables. The Hosmer–Lemeshow goodness of fit test was carried out on the final models to assess if they fitted the observed data adequately. Receiver operating curves with area under the curve (AUC) values were used to show the models’ predictive ability. A *p*-value < 0.05 was considered significant in the final models. The ICC was also calculated for the three viruses.

## 3. Results

### 3.1. Cattle Owner and Herd Information

A total of 34 cattle-owning household heads (28 males; 82.4% and six females; 17.6%) from five locations in the Baringo South sub-County were recruited for this study. The age of the household heads ranged from 26 to 71 years. Only seven (20.6%) had attained tertiary level education (all male), eight (23.5%) had a secondary level of education (all male), 12 (35.3%) a primary level, and seven (20.6%) none at all. Only four (11.8%) were formally employed while the remaining 30 (88.2%) indicated that farming was their sole source of income and livelihood (Table S1) (Supplementary Materials). According to the calculated socio-economic index (SEI) scores [43], 27 (79.4%) were classified as having a high SEI, while the remaining seven (20.6%) were classified in the lower category. Most of the households (n = 20, 58.8%) lived within a 10 km radius of a wildlife conservancy while the remaining 14 (41.2%) lived more than 10 km away from the conservancy (Table S1) (Supplementary Materials).

According to the calculated tropical livestock units (TLUs), only nine (26.5%) herds were above the mean of all the sampled herds, while 25 (73.5%) were below it. The majority of the sampled cattle (n = 383, 95.8%) were of the Boran breed while the remainder were cross-bred (11/400), Friesian (n = 3, 0.8%), and Sahiwal (n = 3, 0.8%) breeds. The majority of the cattle (n = 289, 72.3%) were female while 111 (27.8%) were male. Most of them had not received any vaccine (n = 353, 88.3%) and a few had received vaccination for foot and mouth disease (n = 37, 9.3%), foot and mouth and lumpy skin disease (n = 9, 2.3%), and lumpy skin disease (n = 1, 0.3%). The rectal temperature of all the sampled cattle was within the normal range (36.7–39.1 °C). On inspection during sampling, most of the animals (n = 360, 90%) did not have any ticks. The body condition score of the cattle was measured on a 1–5 scale, with 203 (50.8%) having a score of between 3–5 while the remaining 197 (49.2%) had a score of between 1–2.5 (Table 1, Table 2 and Table 3).

### 3.2. Seroprevalence of RVFV, BTV, and EHDV

From a total of 34 herds tested, 24 herds (70.6%) were seropositive for RVFV, with 62 seropositive animals (15.5%) from a total of 400 cattle samples. A herd was considered positive if at least one animal in the herd was seropositive (Table 4). In total, 366 (91.5%) and 364 (91%) cattle from all 34 herds were seropositive for BTV and EHDV, respectively (Table 4). Dual exposure to BTV and EHDV was detected in 348 (87%) cattle from all tested herds. Dual exposure to BTV and RVFV or RVFV and EHDV was detected in 62 (15.5%) cattle from 24 (70.6%) herds and in 59 (14.8%) cattle from 24 (70.6%) herds, respectively. Triple exposure (RVFV–BTV–EHDV) was detected in 59 (14.8%) cattle from 24 (70.6%) herds (Table 4).

**Figure 2:**
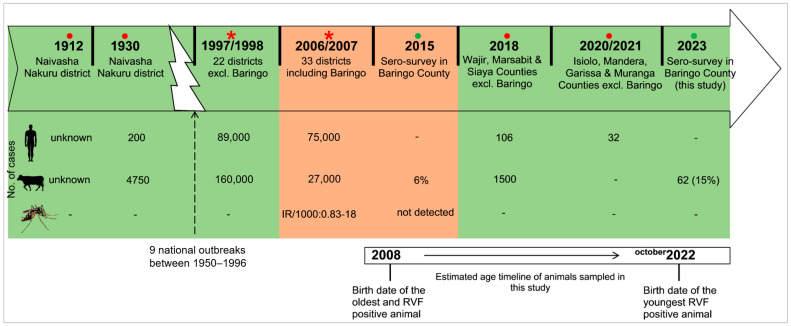
Timeline of the major RVF outbreaks and cross-sectional studies in Kenya in relation to the age of animals sampled in this study. Reports involving Baringo County are highlighted in brown while others are in green. Red circles (●) indicate localized outbreaks [44,45,46,47], red asterisks (*) show national epizootics [7,48], and green circles (●) indicate sero-surveys [34].

The seropositivity of the three viruses according to location is illustrated by bar graphs in Figure 1. The differences were not significant among the five locations. None of the tested animals had IgM antibodies against RVFV. The oldest positive animal that was sampled was approximately born in 2008 (and was about 15 years old at the time of sampling), which was after the latest outbreak in Baringo County in 2006/2007 (Figure 2). After 2006/2007, none of the localized outbreaks occurring in Kenya involved Baringo County. The youngest RVFV-seropositive animal was born in approximately October 2022 and was about 6 months old at the time of sampling (Figure 2).

### 3.3. Risk Factor Analysis of Seropositivity to RVFV, BTV, and EHDV

Results from the univariate logistic regression models show that the age of the cattle owner and the sex, age, and body condition score of the animal were associated with seropositivity to RVFV (Table 1 and Table S1). Therefore, they were included in the RVF multivariate model. With respect to BT, the age of the animal and its body condition score were associated with BTV seropositivity (Table 2) and were included in the BT multivariate model. The age and occupation of the cattle owner and history of RVF in the household were also included in the multivariate analysis for BT because of a *p*-value < 0.2 (Table S1). The age of the animal and its body condition score were associated with seropositivity to EHDV (Table 3) and were included in the EHD multivariate model. The education level of the cattle owner was also included in the multivariate model for EHD because of a *p*-value < 0.2 (Table S1). The rest of the potential risk factors were not associated with seropositivity to either virus and were not included in further analysis.

In the multivariate analysis, the age of the owner was significantly associated with seropositivity to RVFV (*p* = 0.014), with those cattle belonging to household heads in the 30–39 age group being less likely to be seropositive (OR = 0.16, 95% CI = 0.04–0.48, *p* = 0.004) compared to those from heads aged above 60 years (Table 5). Female animals were significantly more likely to be seropositive to RVFV (OR = 5.55, 95% CI = 2.16–18.90, *p* = 0.001) compared to male animals. The age of the animal was also significantly associated with seropositivity to RVFV (*p* = 0.003). Adult cattle were more likely to be seropositive to RVFV (OR = 3.94, 95% CI = 1.35–16.82, *p* = 0.028) compared to calves (Table 5).

The age of the animal was significantly associated with seropositivity to both BTV (*p* < 0.001) and EHDV (*p* < 0.001). Both adult (OR = 180.03, 95% CI = 45.11–1081.51, *p* < 0.001) and young cattle (OR = 6.49, 95% CI = 2.07–25.67, *p* = 0.003) were more likely to be seropositive to BTV compared to calves. Both adult (OR = 26.83, 95% CI = 11.26–71.84, *p* < 0.001) and young cattle (OR = 4.27, 95% CI = 1.55–13.94, *p* = 0.008) were also more likely to be seropositive to EHDV compared to calves (Table 5).

### 3.4. Risk Factor Analysis Based on Viral Dual Exposure

Exposure to each of the tested viruses was considered a potential risk factor in this analysis. The results of this correlation are shown in Table 6. Exposure to BTV was strongly correlated to exposure to EHDV and vice versa (*p* < 0.001). However, RVFV exposure was not significantly correlated to the other two viruses (Table 6). On the other hand, the ICC was calculated as 0.036, 0.039, and 0.04 for RVFV, BTV, and EHDV, respectively.

## 4. Discussion

To advance the knowledge of RVFV circulation during IEPs, the epidemiology of BTV and EHDV, and the associated risk factors, this serosurvey was carried out in Baringo County, Kenya, an RVF high-risk region. We found evidence of past RVFV exposure in cattle based on the presence of IgG antibodies against RVFV. While no IgM antibodies were detected, the presence of IgG antibodies in young cattle (6–12 months) and the fact that all the tested animals were born after 2007 (when the last recorded outbreak occurred in Baringo) indicate the cryptic circulation of RVFV. Furthermore, we report very high seroprevalence rates of BTV and EHDV. From the results of risk factor analysis, the age of the animal was one of the factors associated with exposure to all three viruses. Surveillance of RVF during IEPs helps in understanding its circulation, as it is generally thought to contribute to the maintenance of the virus in vertebrates and its replenishment in mosquito populations [9]. It is also important to define epidemiological zones of other relatively unknown viruses, which in turn informs control efforts against them.

The seroprevalence of RVFV reported here (15.5%) was lower than that previously reported in Tanzania (29.2%) [49] and South Africa (42.9%) [50] during IEP periods. It was, however, higher than the reported seroprevalence in Ijara, Kenya (13.1%) [51] and Kilombero, Tanzania (5.5%) [52]. In the 2006/2007 major outbreak in eastern Africa, both Kenya and Tanzania were affected, with almost similar human and livestock cases reported [53]. In a longitudinal study in Ijara County, higher seroprevalences were reported when migrating herds passed through thick forests compared to those grazing near homesteads. Yet, in our study, the distance of the household/herd from the nearby community wildlife conservancies was not associated with RVFV seropositivity. The presence of IgG antibodies against RVFV indicates previous exposure; however, the oldest animal sampled in this study was 15 years old, implying that all the animals sampled in our study were born after the 2006/2007 major RVF outbreak in East Africa. Thus, it can be assumed that all seropositive animals detected in Baringo County had acquired the virus thereafter. Recent (2021) smaller outbreaks of RVF in Kenya occurred in other counties such as Isiolo, Mandera, Garissa, and Marsabit [47,54], and these counties do not border Baringo. This limits the possibility that the recorded virus circulation was derived from RVFV-infected animal incursions. Given that vaccination against RVFV in this region was last conducted during the 2007 outbreak and maternally derived antibodies decline after 6 months, seropositivity in animals between 6 to 12 months also suggests recent exposure to the virus. Moreover, modeling studies have shown a decline in immunity in cattle within 5 years following an outbreak [55].

The seroprevalence of BTV (91.5%) in this study is similar to other studies in Kenya and beyond, while the EHDV seroprevalence (91%) we recorded is higher than those reported in previous studies in Kenya and elsewhere. For instance, in western Kenya, 94% and 64% of indigenous calves had seroconverted to BTV and EHDV by 51 weeks of age, respectively [21]. In Machakos County, Kenya, 50% of the sampled cattle were seropositive for BTV while in Isiolo County, a seropositivity more similar to our study was reported (86%) [56]. These regional differences might arise due to differences in the ecology, which affects the breeding and activity of the vectors. Elsewhere, varying seroprevalence rates against BTV in cattle were reported, with 99.5% in Mayotte [57], 88.8% in Mali [17], 19.4% in Sudan [58], and 62% in Zimbabwe [59]. In the Campania region of Italy, a prevalence of 45.2% was observed in cattle, while in Pakistan, 66% of the tested cattle were seropositive for BTV [60,61]. Even though in most of the epidemiological studies involving BTV/EHDV, the tested animals do not exhibit any clinical illness, they reflect the importance of cattle in the maintenance of the viruses.

We noted a high seroprevalence of BTV, similar to most of the previous studies, emphasizing a very high infection rate in cattle which is probably driven by the presence and activity of *Culicoides* spp. and subclinically infected cattle in the tested herds. Baringo County is a semi-arid region, hence most of the cattle graze on the shores of Lakes Baringo and Bogoria, around irrigation plots and in swamps in the nearby wildlife conservancies. These areas are perennially wet and muddy, conditions highly conducive for *Culicoides* spp. [62]. Although the high temperatures and drought-like conditions experienced in the county reduce vector survival, they create shallower and warmer water bodies, exposing mud which favors the breeding of midges. Likewise, these conditions also push cattle to aggregate at watering points for most of the day, exposing them to biting midges [63].

Previous studies have shown that the epidemiological features of BTV are likely to mirror those of EHDV as they share the same vector and ecological niche. However, exposure to EHDV is consistently lower than to BTV in most of the studies, underlining subtle differences in the epidemiology of BTV and EHDV. Insignificant differences have been reported in the replication rate, temperature required, and extrinsic incubation period of BTV, EHDV, and African horse sickness virus in *C. sonorensis* [64,65]. We therefore hypothesize that the difference in seroprevalence is a result of specific virus strain–vector species relationships. It is possible that there are differences in vector competence for different orbiviruses in the same vector species and for the same orbivirus in different vector species [64]. A recent entomological survey in Baringo County found *C. imicola* to be the most prevalent species [66], hence relevant information could be generated by serotyping the local BTV and EHDV strains and then using them for transmission experiments in *C. imicola*.

The seroprevalence of EHDV in our survey was lower than that in a cross-sectional study of EHDV in cattle in Mayotte (96.9%), possibly due to the previous outbreaks of both BTV and EHDV in Mayotte [57]. In a case-control study in dairy cattle in Israel, the seroprevalence of EHDV was reported to be 72% in clinically affected cattle while there was also a higher prevalence in subclinical animals in the herd (57%) [67]. In Zimbabwe, a seroprevalence of 57% was reported in cattle towards the end of the rainy season. This seroprevalence was higher than that recorded during the dry season [59]. In summary, several factors including wetland cover, soil type, temperature, livestock management system, vector control, and wind patterns influence the exposure of animals in a given locality, leading to the reported differences in seropositivity to BTV and EHDV [58,68].

There was a significant correlation between BTV and EHDV seropositivity, which was absent between RVFV and each of the two viruses. This dual exposure/co-infection has been described in previous studies and is plausible given that BTV and EHDV usually share the same *Culicoides* vector and transmission ecology [21,57,62]. High seropositivity to these two viruses in cattle in the absence of clinical cases implies an endemic situation in Baringo County and most likely the rest of Kenya. It also signifies natural infection as the Directorate of Veterinary Services in the county confirmed that no vaccination had been carried out against the two viruses (personal communication).

In addition to the loss of body condition, abortions, and decreased milk production associated with a subclinical disease state, other serotypes of EHDV (EHDV serotypes 2, 6, and 7) [24] and BTV (BTV serotype 8) [69] are also known to cause overt clinical signs in cattle and possibly death. Control efforts should therefore be implemented in endemic zones where there is high *Culicoides* spp. activity, such as in Baringo County [66]. These measures generally include vaccination with the dominant BTV strains in the area and keeping livestock off low-lying wet grazing lands. In Kenya, a BLUEVAX™ freeze-dried, live attenuated bluetongue vaccine prepared from the seven common strains is available on the market for yearly sheep vaccination [70]. However, widespread use of the vaccine is hampered by the cost and logistical reasons. On the other hand, vaccination for EHDV is carried out only in Japan and the USA, where the disease causes significant losses in white-tailed deer [24].

Several key variables were investigated that are likely to affect/associate with the seropositivity of the three viruses. The age of the animal was significantly associated with seropositivity to all three viruses, indicating that adult animals were more seropositive compared to young animals/calves. For RVFV, age-dependent exposure has been reported previously and is compounded by the longevity of IgG antibodies in cattle. Therefore, the older the animal, the more chance it has to be infected by the virus [49,71,72]. The same trend is also evident for BTV and EHDV, where older cattle are likely to be more frequently exposed to *Culicoides* bites compared to the young [60,61,73]. We found female animals were significantly more infected with RVFV compared to males. This could be because most of the females stay in the herd longer for reproductive purposes and herd growth. On the other hand, bulls and oxen are usually sold off quickly for slaughter or social ceremonies [71]. With respect to the animal owner, an older age (>60) was associated with seropositivity compared to those between 30–39 years. This is a linkage that has not been investigated in previous studies and could possibly be attributed to better knowledge and livestock management practices in younger compared to older cattle owners.

We also report low ICC values for the three viruses, which can help in the design of future studies. With respect to RVFV, the ICC was lower than that reported in Tana River County, Kenya [35] and in South Africa [50]. This shows that there was less between-herd variation in seroprevalence to RVFV in Baringo County compared to Tana River County and South Africa. A low ICC is reflective of conditions that are more conducive to IEP circulation and widespread exposure. A high ICC therefore implies that more herds should be used during epidemiological studies with fewer animals sampled per herd. Overall, one of the dangers of clustering is a misrepresentation of the prevailing risk of RVF or any other disease [35].

A limitation of our study is that some recruited households had fewer cattle than previously indicated, leading to the recruitment of additional households on sampling day and more than 10 animals being sampled per herd. Additionally, viral neutralization tests were not carried out on the ELISA-positive samples to ascertain the presence of neutralizing antibodies. However, previous studies have shown that the difference between the ELISA test and neutralization tests is minimal, with 95–100% of ELISA-positive samples confirmed to have neutralizing antibodies [49]. The IDvet ELISA kit that we used has been validated and its sensitivity and specificity were reported to be 91–100% and 100%, respectively [39]. While there is a possibility of cross-reaction between the closely related BTV and EHDV, the IDvet ELISA kit that we used showed 100% specificity for EHDV in the presence of BTV in test serum samples [41]. Despite these limitations, we are confident that the seropositivity reported in this study is a true reflection of the exposure to RVFV, BTV, and EHDV in cattle in Baringo County, Kenya.

## 5. Conclusions and Recommendations

Our study highlights post-outbreak exposure to RVFV in Baringo County in northwestern Kenya, coupled with high seroprevalence rates against BTV and EHDV in cattle. The correlation between BTV and EHDV seroprevalence rates implies that effective control initiatives should focus on the two viruses together. Despite the high seroprevalence rates, most of the animals did not have apparent clinical signs, and for those that did, it was not possible to link the symptoms of disease to the viruses investigated. Based on these findings, there is a need to promote awareness of RVFV and other arboviruses in the community, county Directorate of Veterinary Services, and medical officers.

## Figures and Tables

**Table 1:** Individual-level descriptive statistics of cattle (n = 400) in Baringo County and univariate analysis of potential risk factors associated with Rift Valley fever virus.

Risk Factor and Category	Total No. (%)	Rift Valley Fever Virus		
		No. +ve (%)	Odds Ratio (95% CI)	*p*-Value
Sex				
Female	289 (72.3)	58 (20.1)	6.72 (2.67–22.56)	**0.000**
Male	111 (27.8)	4 (3.6)		-
Age				
Adult	300 (75)	58 (19.3)	4.55 (1.61–19.12)	**0.013**
Young	40 (10)	3 (7.5)	0.48 (0.02–3.96)	0.540
Calf	60 (15)	1 (1.7)	1.0	**0.000**
Body condition score				
1–2.5	197 (49.3)	38 (19.3)	1.78 (1.03–3.14)	**0.041**
3–5	203 (50.8)	24 (11.8)	1.0	-

CI = confidence interval. *p*-values less than 0.05 are shown in bold.

**Table 2:** Individual-level descriptive statistics of cattle (n = 400) in Baringo County and univariate analysis of potential risk factors associated with Bluetongue virus.

Risk Factor and Category	Total No. (%)	Bluetongue Virus		
		No. +ve (%)	Odds Ratio (95% CI)	*p*-Value
Sex				
Female	289 (72.3)	266 (92)	1.27 (0.58–2.65)	0.532
Male	111 (27.8)	100 (90.1)	1.0	-
Age				
Adult	300 (75)	297 (99)	61.54 (20.21–268.48)	**0.000**
Young	40 (10)	37 (92.5)	2.49 (1.01–6.64)	0.056
Calf	60 (15)	32 (53.3)	1.0	**0.000**
Body condition score				
1–2.5	197 (49.3)	174 (88.3)	0.43 (0.20–0.90)	**0.028**
3–5	203 (50.8)	192 (94.6)	1.0	-

CI = confidence interval. *p*-values less than 0.05 are shown in bold.

**Table 3:** Individual-level descriptive statistics of cattle (n = 400) in Baringo County and univariate analysis of potential risk factors associated with Epizootic hemorrhagic disease virus.

Risk Factor and Category	Total No. (%)	Epizootic Hemorrhagic Disease Virus		
		No. +ve (%)	Odds Ratio (95% CI)	*p*-Value
Sex				
Female	289 (72.3)	262 (90.7)	0.86 (0.37–1.82)	0.700
Male	111 (27.8)	102 (91.9)	1.0	-
Age				
Adult	300 (75)	293 (97.7)		**0.000**
Young	40 (10)	35 (87.5)	4.67 (1.71–15.09)	**0.005**
Calf	60 (15)	36 (60)	1.0	**0.000**
Body condition score				
1–2.5	197 (49.3)	173 (87.8)	0.45 (0.21–0.92)	**0.032**
3–5	203 (50.8)	191 (94.1)	1.0	-

CI = confidence interval. *p*-values less than 0.05 are shown in bold.

**Table 4:** Individual animal (n = 400) and herd-level (n = 34) seroprevalence of RVFV, BTV, and EHDV in cattle from Baringo County.

Arbovirus	Animal-Level Seroprevalence		Herd-Level Seroprevalence	
	No. of Seropositive Cattle	% Seroprevalence	No. of Seropositive Herds	% Seroprevalence
Rift Valley fever	62	15.5	24	70.6
Bluetongue	366	91.5	34	100
Epizootic hemorrhagic disease	364	91	34	100
Bluetongue–Epizootic hemorrhagic dual exposure	348	87	34	100
Bluetongue–Rift Valley fever dual exposure	62	15.5	24	70.6
Rift Valley fever–Epizootic hemorrhagic disease dual exposure	59	14.8	24	70.6
RVFV–BTV–EHDV triple exposure	59	14.8	24	70.6

RVFV = Rift Valley fever virus, BTV = Bluetongue virus, EHDV = Epizootic hemorrhagic disease virus.

**Table 5:** Multivariable models fitted to the cattle data showing significant predictors for RVFV, BTV, and EHDV seropositivity.

Risk Factor and Category	Rift Valley Fever Virus		Bluetongue Virus		Epizootic Hemorrhagic Disease Virus	
	Adjusted OR (95% CI)	*p*-Value	Adjusted OR (95% CI)	*p*-Value	Adjusted OR (95% CI)	*p*-Value
Age of owner						
19–29	0.83 (0.35–1.87)	0.657	1.23 (0.28–5.85)	0.787		
30–39	0.16 (0.04–0.48)	**0.004**	0.09 (0.02–0.38)	0.002		
40–49	0.66 (0.31–1.39)	0.280	0.6 (0.17–2.04)	0.413		
50–59	0.61 (0.25–1.42)	0.267	3.08 (0.70–16.69)	0.153		
>60	1.0	**0.014**	1.0	0.193		
Sex of animal						
Female	5.55 (2.16–18.90)	**0.001**				
Male	1.0	-				
Age of animal						
Adult	3.94 (1.35–16.82)	**0.028**	180.03 (45.11–1081.51)	**0.000**	26.83 (11.26–71.84)	**0.000**
Young	0.55 (0.03–4.62)	0.618	6.49 (2.07–25.67)	**0.003**	4.27 (1.55–13.94)	**0.008**
Calf	1.0	**0.003**	1.0	**0.000**	1.0	**0.000**
BCS						
1–2.5					0.51 (0.22–1.13)	0.103
3–5					1.0	-

OR = odds ratio, CI = confidence interval, BCS = body condition score, GVIF = generalized variance inflation factors, AUC = area under curve, RVFV = Rift Valley fever virus, BTV = Bluetongue virus, EHDV = Epizootic hemorrhagic disease virus. *p*-values less than 0.05 in the adjusted model are shown in bold. The independent variables had GVIFs < 1.2 for the three models, indicating the absence of serious multicollinearity. The Hosmer–Lemeshow goodness of fit test produced chi-square values of 4.0376, df = 8, *p*-value = 0.8537 for RVFV, 2.8903, df = 8, *p*-value = 0.9411 for BTV, and 1.3926, df = 8, *p*-value = 0.9944 for EHDV, suggesting that the three models fit the data well. The AUC for the three models was 0.75 (RVFV), 0.75 (BTV), and 0.84 (EHDV).

**Table 6:** Correlation analysis based on virus seropositivity in cattle in Baringo County.

Target Virus	Factors	No. Tested	No. +ve (%)	OR (95% CI)	*p*-Value
Rift Valley fever					
	BTV+	366	62 (16.9)	-	-
	BTV−	34	0 (0)	-	
	EHDV+	364	59 (16.2)	2.13 (0.73–9.05)	0.223
	EHDV−	36	3 (8.3)	-	
**Bluetongue**					
	EHDV+	364	348 (95.6)	21.75 (9.64–50.48)	**0.000**
	EHDV−	36	18 (50)	-	
	RVFV+	62	62 (100)	1.293440 × 10^7^ (2.53 × 10^−11^ 6.81 × 10^112^)	0.984
	RVFV−	338	304 (89.9)	-	

CI = confidence interval, OR = odds ratio, BTV+/− = Bluetongue virus seropositive/negative, EHDV+/− = Epizootic hemorrhagic disease virus seropositive/negative, RVFV+/− = Rift Valley fever virus seropositive/negative. *p*-values less than 0.05 are shown in bold.

## Data Availability

The original data presented in the study are openly available in the University of Liverpool, Research Data Catalogue repository at https://doi.org/10.17638/datacat.liverpool.ac.uk/2528.

## Supplementary Materials

The following supporting information can be downloaded at: https://www.mdpi.com/article/10.3390/pathogens13080613/s1, Table S1: Univariate analysis of owner/herd-related potential risk factors associated with RVFV, BTV, and EHDV seropositivity. Supplementary File S1: Blood collection questionnaire. Supplementary File S2: Arbovirus survey questionnaire.
