# Supplementary material for: Exposure to Arboviruses in Cattle: Seroprevalence of Rift Valley Fever, Bluetongue, and Epizootic Hemorrhagic Disease Viruses and Risk Factors in Baringo County, Kenya
Source: Pathogens. 2024 Jul 24;13(8):613. doi: 10.3390/pathogens13080613 (PMC11357150; doi:10.3390/pathogens13080613)
Supplement: Supplementary file 1 [file pathogens-13-00613-s001.zip › File S1- Blood collection questionnaire.pdf]

Blood_collection_questionnaire

Name of owner

Enter full name(firstname, surname)

Gender of the owner

- ○ Female
- ○ Male

Age of owner

If exact age is unknown enter an estimate

Tribe of the owner

Animal ID

Sex of the animal

- ○ Female
- ○ Male

Age of the animal

Breed of the animal

Has this animal been vaccinated before?

- ○ Yes
- ○ No

What was it vaccinated against?

Has this animal been sick before?

- ○ Yes
- ○ No

What were the clinical signs?

Temperature

____

Mucous membranes

- ◯ Normal
- ◯ Pale
- ◯ Yellow
- ◯ Red
- ◯ Purple

Body condition score

____

Are they any ticks present on the animal?

- ◯ Yes
- ◯ No
