# Supplementary material for: Exposure to Arboviruses in Cattle: Seroprevalence of Rift Valley Fever, Bluetongue, and Epizootic Hemorrhagic Disease Viruses and Risk Factors in Baringo County, Kenya
Source: Pathogens. 2024 Jul 24;13(8):613. doi: 10.3390/pathogens13080613 (PMC11357150; doi:10.3390/pathogens13080613)
Supplement: Supplementary file 1 [file pathogens-13-00613-s001.zip › Table S1.pdf]

**Table S1:** Univariate analysis of owner/herd related potential risk factors associated with RVFV, BTV and EHDV seropositivity.

| Risk factor and category | Total No. (%) | Rift Valley fever virus | | | Bluetongue virus | | | Epizootic hemorrhagic disease virus | | |
|---|---|---|---|---|---|---|---|---|---|---|
| | | No. +ve (%) | OR (95% CI) | p-value | No. +ve (%) | OR (95% CI) | p-value | No. +ve (%) | OR (95% CI) | p-value |
| **Owner variables** | | | | | | | | | | |
| *Sex* | | | | | | | | | | |
| Female | 72 (18) | 12 (16.7) | 1.11 (0.54-2.15) | 0.763 | 67 (93.1) | 1.3 (0.53-3.93) | 0.602 | 68 (94.4) | 1.84 (0.70-6.32) | 0.266 |
| Male | 328 (82) | 50 (15.2) | 1.0 | - | 299 (91.2) | 1.0 | - | 296 (90.2) | | - |
| *Age* | | | | | | | | | | |
| 19-29 | 56 (14) | 11 (19.6) | 0.87 (0.38-1.89) | 0.732 | 52 (92.9) | 0.85 (0.25-3.37) | 0.803 | 51 (91.1) | 0.67 (0.20-2.35) | 0.507 |
| 30-39 | 67 (16.8) | 3 (4.5) | 0.17 (0.04-0.50) | **0.005** | 57 (85.1) | 0.37 (0.13-1.02) | 0.058 | 59 (88.1) | 0.48 (0.16-1.41) | 0.179 |
| 40-49 | 96 (24) | 14 (14.6) | 0.61 (0.29-1.23) | 0.175 | 86 (89.6) | 0.56 (0.20-1.53) | 0.263 | 88 (91.7) | 0.72 (0.24-2.08) | 0.540 |
| 50-59 | 67 (16.8) | 9 (13.4) | 0.55 (0.23-1.23) | 0.161 | 64 (95.5) | 1.40 (0.37-6.65) | 0.638 | 59 (88.1) | 0.48 (0.16-1.41) | 0.179 |
| > 60 | 114 (28.5) | 25 (21.9) | 1.0 | **0.014** | 107 (93.9) | 1.0 | 0.193 | 107 (93.9) | 1.0 | 0.624 |
| *Education* | | | | | | | | | | |
| Tertiary | 92 (23) | 14 (15.2) | 0.7 (0.31-1.54) | 0.368 | 85 (92.4) | 0.83 (0.24-2.72) | 0.761 | 83 (90.2) | 0.5 (0.13-1.60) | 0.263 |
| Secondary | 115 (28.8) | 14 (12.2) | 0.54 (0.24-1.18) | 0.120 | 102 (88.7) | 0.54 (0.17-1.49) | 0.257 | 99 (86.1) | 0.33 (0.09-0.96) | 0.059 |
| Primary | 115 (28.8) | 18 (15.7) | 0.72 (0.34-1.53) | 0386 | 106 (92.2) | 0.81 (0.24-2.43) | 0.710 | 108 (93.9) | 0.83 (0.21-2.86) | 0.778 |
| None | 78 (19.5) | 16 (20.5) | 1.0 | 0.489 | 73 (93.6) | 1.0 | 0.636 | 74 (94.9) | 1.0 | 0.111 |
| *Occupation* | | | | | | | | | | |
| Salaried employment | 43 (10.8) | 7 (16.3) | 1.07 (0.42-2.39) | 0.881 | 42 (97.7) | 4.28 (0.88-77.04) | 0.157 | 40 (93) | 1.36 (0.46-5.82) | 0.625 |
| Farming | 357 (89.3) | 55 (15.4) | 1.0 | - | 324 (90.8) | 1.0 | - | 324 (90.8) | 1.0 | - |
| *SEI* | | | | | | | | | | |
| Low | 63 (15.8) | 10 (15.9) | 1.03 (0.47-2.09) | 0.929 | 56 (88.9) | 0.70 (0.30-1.81) | 0.42 | 59 (93.7) | 1.55 (0.59-5.34) | 0.426 |
| High | 337 (84.3) | 52 (15.4) | 1.0 | - | 310 (92) | 1.0 | - | 305 (90.5) | 1.0 | - |

| | | | | | | | | | |
|---|---|---|---|---|---|---|---|---|---|
| *TLUs* | | | | | | | | | |
| Low | 271 (67.8) | 45 (16.6) | 1.31 (0.73-2.45) | 0.377 | 247 (91.1) | 0.86 (0.38-1.82) | 0.711 | 250 (92.3) | 1.57 (0.77-3.13) | 0.208 |
| High | 129 (32.3) | 17 (13.2) | 1.0 | - | 119 (92.2) | 1.0 | - | 114 (88.4) | 1.0 | - |
| *History of RVF* | | | | | | | | | | |
| Yes | 83 (20.8) | 14 (16.9) | 1.14 (0.57-2.13) | 0.699 | 79 (95.2) | 2.06 (0.79-7.10) | 0.185 | 78 (94) | 1.70 (0.69-5.08) | 0.292 |
| No | 317 (79.3) | 48 (15.1) | 1.0 | - | 287 (90.5) | 1.0 | - | 286 (90.2) | 1.0 | - |
| *Knowledge of RVF* | | | | | | | | | | |
| High | 72 (18) | 10 (13.9) | 0.86 (0.39-1.71) | 0.677 | 67 (93.1) | 1.30 (0.53-3.93) | 0.602 | 64 (88.9) | 0.75 (0.34-1.82) | 0.491 |
| Low | 328 (82) | 52 (15.9) | 1.0 | - | 299 (91.2) | 1.0 | - | 300 (91.5) | 1.0 | - |
| *Distance to conservancy* | | | | | | | | | | |
| 1-10 km | 240 (60) | 34 (14.2) | 0.78 (0.45-1.35) | 0.368 | 220 (91.7) | 1.05 (0.51-2.14) | 0.884 | 216 (90) | 0.73 (0.34-1.48) | 0.394 |
| >10km | 160 (40) | 28 (17.5) | 1.0 | - | 146 (91.3) | 1.0 | - | 148 (92.5) | 1.0 | - |

OR = odds ratio, CI = confidence interval. BCS = body condition score, TLU = tropical livestock units, SEI = socio-economic index, -RVF = Rift Valley fever, p-values less than 0.05 are shown in bold.
